# Trends in food allergy among Hong Kong preschoolers: Findings from 2006, 2013, and 2020 surveys

**DOI:** 10.1111/pai.70188

**Published:** 2025-09-01

**Authors:** Agnes S. Y. Leung, Oi Man Chan, Noelle Anne Ngai, Hing Yee Sy, Ann W. S. Au, Rosetta T. C. Leung, Jaimie Y. M. Ngai, Edmund Yung, Man Fung Tang, Gary W. K. Wong, Ting Fan Leung

**Affiliations:** ^1^ Department of Paediatrics Prince of Wales Hospital, The Chinese University of Hong Kong Hong Kong China; ^2^ Hong Kong Hub of Paediatric Excellence The Chinese University of Hong Kong Hong Kong China; ^3^ School of Nursing and Health Sciences Hong Kong Metropolitan University Hong Kong China

**Keywords:** atopic conditions, Chinese population, doctor‐diagnosed allergies, epidemiological trends, food allergy prevalence, Hong Kong, longitudinal survey, parent‐reported allergies, preschool children, urban health

## Abstract

**Background:**

Food allergy (FA) significantly impacts quality of life and public health, but data on prevalence trends in Asia remain limited. This study investigated trends in FA prevalence and related allergic conditions among preschool Chinese children over a 15‐year period.

**Methodology:**

Cross‐sectional surveys were conducted in 2006, 2013, and 2020, targeting nurseries, preschool, and daycare centers across Hong Kong. Data were collected using a standardized, parent‐reported questionnaire adapted from the International Study of Asthma and Allergy in Childhood (ISAAC), assessing the prevalence of perceived (parent‐reported) and probable (doctor‐diagnosed) FA.

**Results:**

A total of 11,537 Chinese children aged 2–7 years from 70 schools were surveyed across the three time points, with response rates ranging from 60% to 80%. The prevalence of perceived FA increased from 6.1% (95% CI, 5.4%–6.9%) in 2006 to 9.5% (95% CI, 8.6%–10.5%) in 2013 but declined to 8.5% (95% CI, 7.7%–9.3%) in 2020. Probable FA prevalence showed nonsignificant change, from 4.4% (95% CI, 3.8%–5.1%) in 2006 to 5.1% (95% CI, 4.5%–5.8%) in 2020. Longitudinal analysis revealed significant sustained increases in egg (doubled to 1.86%), peanut (nearly tripled to 1.52%), tree nut (five‐fold increase to 0.68%), and shellfish allergic reactions (increased to 1.99%). Trends in atopic comorbidities included declines in asthma and wheezing, fluctuations in rhinitis, and an increase in eczema.

**Conclusions:**

Perceived FA increased over time, while probable FA remained stable, with shifts in allergens. Further research should explore this divergence and focus on enhancing allergy services and improving public awareness.

**Abbreviations**
AFR: adverse food reactions
aOR: adjusted odds ratio
CIs: confidence intervals
FA: food allergy
HK: Hong Kong
ISAAC: International Study of Asthma and Allergy in Childhood
ORs: odds ratios

**Key message**

This 15‐year population‐based study reveals a divergence between perceived and diagnosed food allergies among Hong Kong preschool children, with parent‐reported cases increasing while doctor‐diagnosed cases remain stable. The study identifies a sustained increase in allergic reactions to key allergens, including egg, peanut, tree nut, and shellfish. These findings underscore the urgent need to address discrepancies in allergy diagnosis and enhance public health strategies to ensure better healthcare access and public education in Asian cities.

## 1 INTRODUCTION

Food allergy (FA) is an increasingly significant global health concern, particularly in childhood, where it can lead to a wide range of adverse food reactions (AFRs), from mild to life‐threatening conditions such as anaphylaxis.^1^ The early onset of FA, which often presents in infancy, presents unique challenges for children and their families. A cohort study in Australia revealed a 10% prevalence of challenge‐proven FA in 1 year olds.^2^ Affected children must manage their condition from a young age, which can lead to developmental and psychosocial difficulties.^3^ Furthermore, the constant risk of severe allergic reactions, including anaphylaxis, introduces an acute and unpredictable medical emergency, placing additional stress on both patients and caregivers.^4^

National data from the United Kingdom indicates a sharp increase in food anaphylaxis admissions, rising from 2.1 to 9.2 per 100,000 children under 15 years from 1998 to 2018.^5^ Similarly, the United States experienced a 200% increase in food‐induced anaphylaxis‐related emergency visits among children aged 5–17 between 2005 and 2014.^6^ In Hong Kong (HK), we have previously reported a rise in anaphylaxis cases from 2009 to 2019 using a public healthcare database, with particularly high rates observed in children (2019 rates: 3.51 vs. 1.82 for adults).^7^ This increasing trend underscores the growing burden of FA and the urgent need for continued attention in both clinical and public health contexts.^8^

While the rising incidence of anaphylaxis is well‐documented, understanding the prevalence of FA is equally essential for assessing the broader societal impact. Anaphylaxis incidence captures the acute, immediate risk of severe allergic reactions; however, FA prevalence offers critical insight into the long‐term, chronic burden on individuals and healthcare systems. To address the expanding burden of FA effectively, it is crucial to integrate both prevalence data, which track long‐term trends in diagnoses, and anaphylaxis incidence data, which highlight the frequency of severe reactions. This balanced approach will ensure more comprehensive strategies for FA prevention, diagnosis, and management.

To address this gap, we conducted three cross‐sectional surveys in China in 2006, 2013, and 2020, using consistent methodology and parent‐proxy reports, following the exact same approach as the 2006 survey.^9^ This study aimed to provide a detailed analysis of trends in the prevalence of FA and related allergic disorders and the clinical profiles of food‐allergic preschool‐aged children over the past 15 years, offering invaluable insights for clinicians, researchers, and policymakers, particularly in the context of the East.

## 2 PATIENTS AND METHODS

Population‐based surveys utilizing consistent methodologies were conducted at three time points: 2006–2007, 2013–2014, and 2020–2021, targeting a sample of Chinese nursery and kindergarten students. Informed consent was obtained from both the school principals and the participants' parents. The study activities were approved by the Joint Chinese University of Hong Kong—New Territories East Cluster Clinical Research Ethics Committee.

### 2.1 Survey development

The current parent‐report survey builds upon our 2006 study, which included items adapted from the Chinese version of the International Study of Asthma and Allergy in Childhood (ISAAC) questionnaire, originally developed and validated by pediatricians and pediatric allergists.^9^, ^10^ The survey collected information on participants' demographics, allergic respiratory symptoms, other allergic disorders, and potential atopy‐related risk factors.

### 2.2 Study population

Eligible participants for the study were Chinese preschool children aged 2–7 years who were enrolled in nurseries and kindergartens in HK. We selected ages 2–7 to capture both early‐onset allergic manifestations typically appearing in the first 2–3 years of life and the critical transition period where transient allergies (like milk or egg) that often resolve, while persistent allergies (such as peanut or shellfish) stabilize. Partnering with nurseries (2–3 years) and kindergartens (3–6 years) in our region, we created an efficient sampling framework for centralized questionnaire distribution, boosting participation, and minimizing logistical issues. For each cross‐sectional study, comprehensive lists of all preschool institutions were obtained from the Education Bureau of the Hong Kong Special Administrative Region. The nurseries and kindergartens were categorized into four geographical regions: Hong Kong Island, Kowloon, New Territories East, and New Territories West, and were randomly selected to achieve the target sample size in proportion to the preschool populations residing in each of these four regions according to HK Census data (sampling details in Appendix S1).

### 2.3 Outcome measures

We classified FA based on two definitions: perceived food allergy (parent‐reported AFR occurring either in the child's lifetime or past 12 months); and probable food allergy (parent‐reported physician‐diagnosed AFR in the child's lifetime) (details of definition in Appendix S1). For perceived allergies, parents identified specific trigger foods from a comprehensive 32‐item list and answered follow‐up questions about avoidance practices, severity, and reaction frequency. Probable allergies were based on parent‐reported physician diagnoses, but our questionnaire was unable to verify whether physician diagnoses were based solely on parental symptom reports or included objective testing (e.g., IgE sensitization and oral food challenges). Additionally, we assessed the prevalence of other allergic conditions, including asthma, allergic rhinitis, and eczema, as defined in previous research.^10^ Furthermore, we examined food‐related allergic symptoms and potential determinants of FA within the studied population.

### 2.4 Statistical analysis

Children with and without a history of AFR were characterized by frequency (percentages [%]). Bivariate comparisons, using *t*‐tests and chi‐square tests as appropriate, assessed similarities and differences between those with and without AFR. For longitudinal comparisons of proportions (e.g., prevalence of FA across three time points), pairwise independent sample proportion tests (*z*‐tests) were applied. A multivariable logistic regression model was employed to examine associations between AFR history and clinical factors, atopic comorbidities, and socioeconomic factors. Associations are reported as odds ratios (ORs) with 95% confidence intervals (CIs). Multivariable regression analyses were conducted for factors with *p* < .1 in univariate tests. Two‐sided hypothesis tests were applied, with a significance threshold set at *p* < .05. Statistical analyses were performed using SPSS 25.0 (Armonk, NY: IBM Corp.), and figures were generated with GraphPad Prism.

## 3 RESULTS

### 3.1 Time‐trend prevalence of allergic conditions

**FIGURE 1:**
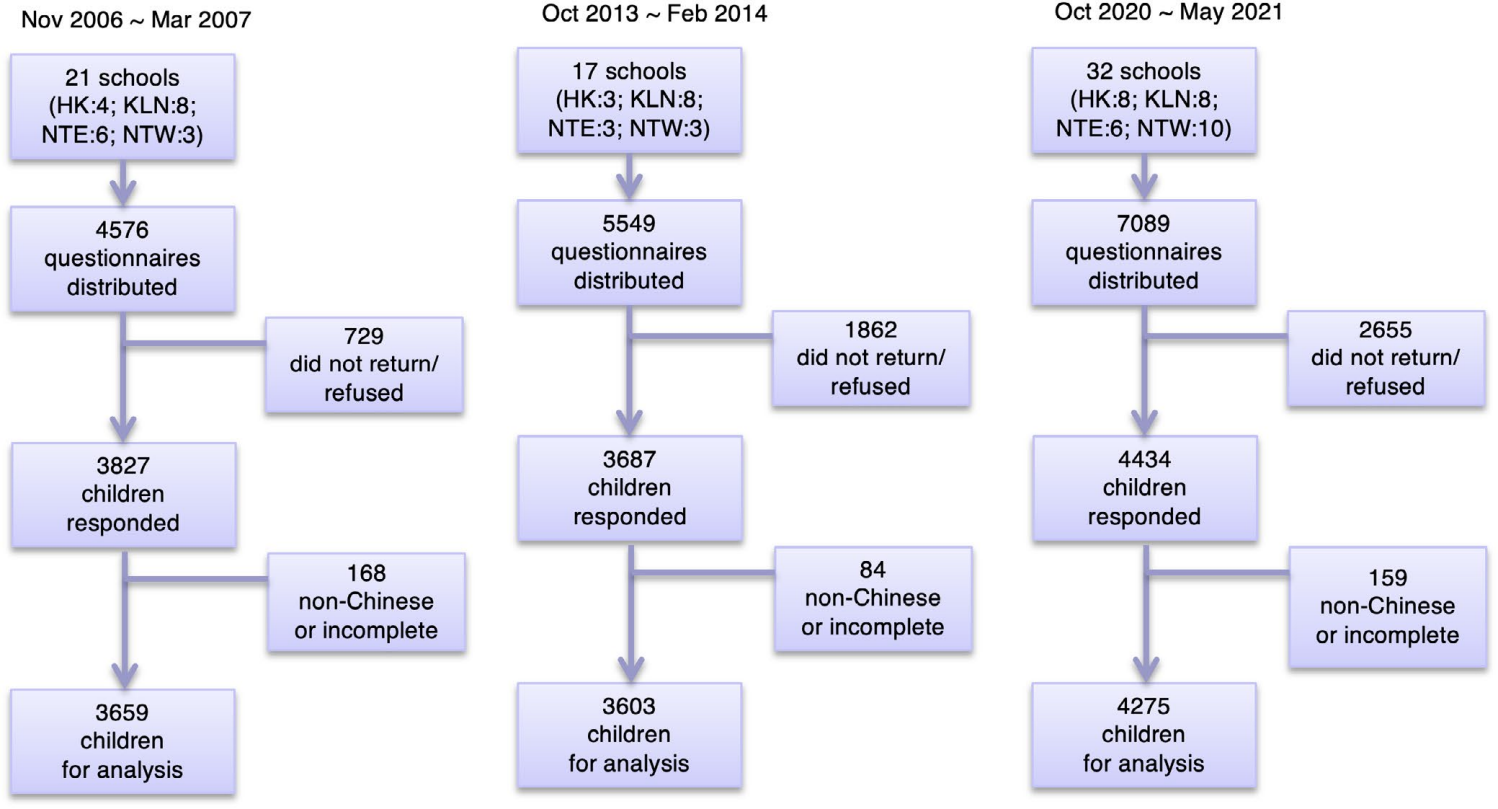
Study participant flow for the 2006, 2013, and 2020 surveys. A total of 70 nurseries and kindergartens participated, and data were collected from 11,537 children across the three time points.

A total of 70 nurseries and kindergartens participated in the study, with participation rates of 83.6% (*n* = 3827), 66.4% (*n* = 3687), and 62.5% (*n* = 4434) for the years 2006, 2013, and 2020, respectively. Figure 1 illustrates the number of schools and participants who were invited, enrolled, and completed the study. Figure S1 illustrates that respondent percentages closely aligned with the population distribution of children in each geographic region across the respective census years. The 2006 survey was conducted from November 2006 to March 2007 (*n* = 4576), the 2013 survey took place from October 2013 to February 2014 (*n* = 5549), and the 2020 survey was carried out between October 2020 and May 2021 (*n* = 7089). Of the returned surveys, only those with complete responses from parents of Chinese ethnicity were included in the analysis, resulting in 3659, 3603, and 4275 usable data for the years 2006, 2013, and 2020, respectively. The study population remained predominantly Chinese (95.6%–97.7%) with balanced gender and age distribution across all three phases (2006–2021) (Table S6). The most notable demographic shift was the increase in parental university education, rising from 12.6% to 44.9% for mothers and 15.8% to 44.0% for fathers between 2006 and 2020.

**FIGURE 2:**
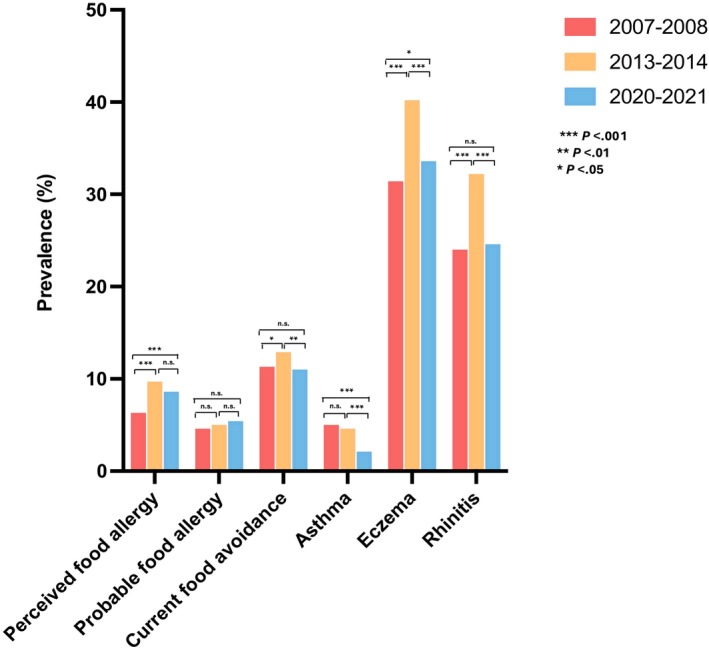
Temporal trends in allergic conditions among Chinese preschool children. Perceived food allergy prevalence increased significantly from 6.1% in 2006–2007 to 9.5% in 2013–2014, before slightly decreasing to 8.5% in 2020–2021, remaining higher than initial rates (*p* < .001). The prevalence of probable food allergy and current food avoidance remained stable over time. Asthma and wheezing illnesses showed significant reductions by 2020–2021. Rhinitis prevalence remained relatively unchanged throughout the study period.

Our analysis highlights temporal trends in allergic conditions among Chinese preschool children, showing notable increases in perceived food allergy and eczema, while the prevalence of asthma and wheezing illnesses decreased over time. Meanwhile, the prevalence of probable food allergy, current food avoidance, and rhinitis has remained relatively stable over the years. The prevalence of perceived food allergy among Chinese children increased significantly, from 6.1% (95% CI, 5.4%–6.9%) in 2006–2007 to 9.5% (95% CI, 8.6%–10.5%) in 2013–2014 (*p* < .001). However, this decreased slightly to 8.5% (95% CI, 7.7%–9.3%) in 2020–2021, while still remaining significantly higher than the initial survey (*p* < .001). For probable food allergy, the prevalence was relatively stable, increasing from 4.4% (95% CI, 3.8%–5.1%) in 2006–2007 to 4.9% (95% CI, 4.2%–5.6%) in 2013–2014 (*p* = .385) and 5.1% (95% CI, 4.5%–5.8%) in 2020–2021 (*p* = .635) (Figure 2). Allergic reaction frequency in the past 12 months remained stable across phases (*p* > .05) (Table S1). Current food avoidance also remained fairly consistent, with prevalence rates of 11.3% (95% CI, 10.3%–12.3%) in 2006–2007, 12.9% (95% CI, 11.9%–14.0%) in 2013–2014 (*p* = .028), and 11.0% (95% CI, 10.1%–11.9%) in 2020–2021 (*p* = .332).

For atopic comorbidities, asthma prevalence showed a significant decline over time, from 5.0% (95% CI, 4.3%–5.7%) in 2006–2007 to 4.6% (95% CI, 3.9%–5.3%) in 2013–2014 (*p* = .442) and further to 2.1% (95% CI, 1.7%–2.6%) in 2020–2021 (*p* < .001). A similar trend was observed for wheeze in the past 12 months, with prevalence increasing from 8.1% (95% CI, 7.3%–9.0%) in 2006–2007 to 13.9% (95% CI, 12.8%–15.1%) in 2013–2014 (*p* < .001) but then dropping to 3.1% (95% CI, 2.6%–3.6%) in 2020–2021 (*p* < .001) (Table S1).

Rhinitis prevalence increased significantly from 24.0% (95% CI, 22.7%–25.4%) in 2006–2007 to 32.2% (95% CI, 30.7%–33.7%) in 2013–2014 (*p* < .001) but remained stable at 24.6% (95% CI, 23.4%–25.9%) in 2020–2021 (*p* = .264). Lastly, eczema prevalence rose significantly from 31.4% (95% CI, 29.9%–32.9%) in 2006–2007 to 40.2% (95% CI, 38.6%–41.8%) in 2013–2014 (*p* < .001) before slightly decreasing to 33.6% (95% CI, 32.3%–35.0%) in 2020–2021 (*p* = .014). The changing prevalence of atopic comorbidities in children over time mirrors that observed in their parents. (Table S2).

### 3.2 Temporal analysis of food allergen patterns

**FIGURE 3:**
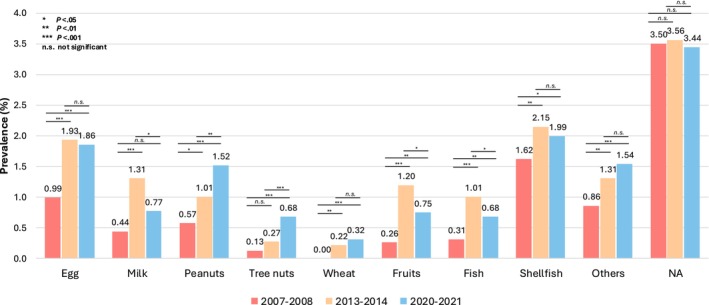
Temporal trends in reported food allergic reactions by allergen triggers (egg, milk, peanuts, tree nuts, wheat, fruits, fish, shellfish, others, and NA) among Chinese preschool children (2006–2007, 2013–2014, and 2020–2021). NA, not available/unknown.

Among the food allergens (combined perceived and probable food allergies), the most pronounced sustained increases were observed for egg allergy, which doubled from 0.99% to 1.86% (*p* < .001), and peanut allergy, which exhibited a steady progression from 0.57% to 1.52% with significant increases across all pairwise comparisons (*p* < .05) (Figure 3). Tree nut allergy demonstrated a marked five‐fold increase from 0.13% to 0.68%, particularly accelerating between 2013 and 2020 (*p* < .001). Shellfish allergy similarly showed significant growth from 1.62% to 1.99% (*p* = .001 between 2006 and 2013). In contrast, several allergens exhibited nonlinear patterns with peak prevalence in 2013, including milk (1.31%, *p* < .001 vs. 2006), fruits (1.20%, *p* < .001 vs. 2006), and fish (1.01%, *p* < .001 vs. 2006), all subsequently declining in 2020. Wheat emerged as a newly reported allergen in 2013 (0.22%, *p* = .003 vs. 2006) with a continued upward trajectory in 2020 (0.32%, *p* < .001 vs. 2013). Unclassified allergies maintained stability across all timepoints (~3.5%, *p* > .05). These findings demonstrate heterogeneous temporal dynamics across major food allergens, with particularly concerning upward trajectories for egg, peanut, tree nut, and shellfish allergies. Table S5 demonstrates differences between perceived and probable food allergy subgroups, with shellfish consistently showing higher perceived rates in both 2006 (21.2% vs. 16.1%, *p* = .046) and 2013 (16.0% vs. 11.5%, *p* = .046). In contrast, no significant differences were observed between perceived and probable rates for other allergens, including egg, milk, peanuts, tree nuts, wheat, or fish.

Analysis of food allergen patterns across early childhood (ages 2–6 years) reveals distinct developmental trajectories (Figure S2). Egg allergic reactions peaked at 21.9% among 2 years olds before declining to 8.8% by age 5, while shellfish reactions showed a progressive increase from 15.2% at age 2 to 21.3% by age 6. Peanut reactions exhibited a mid‐childhood peak (9.4% at ages 4–5), whereas milk reactions remained relatively stable (7.9%–6.6%). Tree nuts, wheat, and fruits demonstrated fluctuating patterns without clear age‐dependent trajectories. These heterogeneous patterns likely reflect age‐related exposure differences, differential resolution rates, and varying diagnostic practices across food categories. Skin reactions and swelling remained the most commonly reported allergic symptoms. Swelling increased from 16.1% to 23.7% (perceived) and 24.5% to 31.7% (probable). Respiratory symptoms, particularly cough, doubled in probable cases (6.4%–9.8%), while severe reactions like shock remained low but slightly increased (2.1%–2.4% for probable cases) (Table S4).

### 3.3 Comparative characteristics of children with and without food allergies

Atopic comorbidities were significantly more common among children with perceived food allergy compared to those without: asthma (7.7% vs. 3.4%), rhinitis (41.4% vs. 25.4%), and eczema (64.2% vs. 32.6%) (all *p* < .001) (Table 1). FA‐associated sociodemographic factors included being Hong Kong‐born (95.7% vs. 93.9%, *p* = .026) and having fewer younger siblings (52.5% vs. 57.3%, *p* = .002). Among those not born in HK, the majority were born in Mainland China. Gender and age distributions were comparable between groups (*p* > .05).

**TABLE 1:** Comparative characteristics of preschool children with food allergies and those without, including demographic, clinical, and atopic comorbidity data.

Population‐weight frequency % (95% CI)	All	Parent‐reported food allergies^a^	No reported food allergies	*p* Value
		*n* = 957	*N* = 10,640	
Perceived food allergy (Parent‐reported AFR)	8 (7.5–8.5)	NA	NA	
Probable food allergy (Parent‐reported doctor‐diagnosed AFR)	4.8 (4.4–5.2)	NA	NA	
Atopic comorbidities				
Asthma	3.8 (3.5–4.2)	7.7 (6.2–9.6)	3.4 (3.1–3.8)	**<.001**
Wheeze ever	15.6 (14.9–16.3)	23.5 (20.9–26.3)	14.9 (14.2–15.6)	**<.001**
Wheeze in past 12 m	8.0 (7.5–8.5)	13.6 (11.5–15.9)	7.4 (6.9–7.9)	**<.001**
Rhinitis	27.2 (26.4–28)	41.4 (38.3–44.5)	25.4 (24.6–26.3)	**<.001**
Eczema	35.4 (34.5–36.3)	64.2 (61.1–67.2)	32.6 (31.7–33.5)	**<.001**
Gender				
Male	51.1 (50.2–52.1)	54.0 (50.8–57.2)	51.0 (50.0–52.0)	.419
Age, years				
<3	8.4 (7.9–8.9)	8.3 (7.8–8.9)	9.7 (7.9–11.7)	.151
> = 3 to <4	30.2 (29.4–31.1)	30.4 (29.6–31.4)	29.4 (26.5–32.4)	.509
> = 4 to <5	30.2 (29.3–31.0)	30.1 (29.2–31.0)	30.1 (27.3–33.1)	.993
> = 5 to <6	26.7 (25.9–27.5)	26.6 (25.7–27.4)	26.4 (23.6–29.3)	.895
> = 6 to <7	4.5 (4.1–4.9)	4.5 (4.1–4.9)	4.4 (3.2–5.9)	.897
Place of birth				
Hong Kong	93.9 (93.5–94.3)	95.7 (94.3–96.9)	93.9 (93.5–94.4)	.**026**
Presence of siblings				
Older	51.2 (50.3–52.2)	49.3 (46.2–52.5)	51.6 (50.7–52.6)	.130
Younger	56.8 (55.9–57.7)	52.5 (49.3–55.6)	57.3 (56.4–58.2)	.**002**
Mother's education level				
Bachelor's degree and above	33.9 (33.0–34.7)	43.2 (40.0–46.3)	33.5 (32.6–34.4)	**<.001**
Father's education level				
Bachelor's degree and above	35.2 (34.3–36.0)	40.2 (37.2–43.4)	35.1 (34.2–36.0)	**<.001**
Maternal allergy history				
Asthma	4.1 (3.8–4.5)	6.7 (5.2–8.4)	3.9 (3.6–4.3)	**<.001**
Rhinitis	26 (25.3–26.8)	35.2 (32.2–38.3)	25.4 (24.6–26.2)	**<.001**
Eczema	12.4 (11.8–13.0)	20.7 (18.2–23.3)	11.7 (11.1–12.3)	**<.001**
AFR	6.9 (6.5–7.4)	15.6 (13.4–18.0)	6.2 (5.7–6.6)	**<.001**
Paternal allergy history				
Asthma	4.7 (4.3–5.1)	7.7 (6.2–9.6)	4.4 (4.1–4.8)	**<.001**
Rhinitis	29.5 (28.7–30.4)	40.6 (37.6–43.8)	28.8 (27.9–29.7)	**<.001**
Eczema	23.7 (22.9–24.4)	28.4 (25.6–31.3)	23.3 (22.5–24.1)	**<.001**
AFR	5.5 (5.1–5.9)	11.2 (9.3–13.3)	5 (4.6–5.4)	**<.001**
Pet ownership				
Dog	17.8 (17.1–18.5)	19 (16.6–21.6)	17.8 (17.0–18.5)	.328
Cat	4.5 (4.1–4.8)	4.4 (3.2–5.8)	4.5 (4.1–4.9)	.871

*Note*: The significant values are bolded.

^a^ Parent‐reported food allergy category includes both perceived and probable cases.

Parental education levels rose alongside FA prevalence: mothers without university degrees declined from 77.2% (2006–2007) to 50.1% (2020–2021), with similar trends for fathers (75.4% to 54.8%, both *p* < .001). Family allergy histories (asthma, rhinitis, eczema, and AFR) were more prevalent in FA children (*p* < .001). Pet ownership showed no association with FA (*p* = .328–.871).

Multivariate analyses in Table 2 showed that maternal education (≥bachelor's degree) was significantly associated with perceived food allergy (aOR: 1.70, 95% CI: 1.32–2.17, *p* < .001), whereas paternal education showed no association (aOR: 1.04, *p* = .773). Children with asthma, rhinitis, or eczema had reduced FA odds, most notably with eczema (aOR: 0.28, *p* < .001). Parental history of AFR lowered perceived FA risk (*p* < .001), though paternal eczema increased it (aOR: 1.41, *p* = .026). Other parental allergies (asthma and rhinitis) and maternal allergic conditions were nonsignificant (Table S3). Environmental factors (nursery attendance, breastfeeding, and pet ownership) and parental smoking (prenatal/current) showed no significant links to FA in adjusted models.

**TABLE 2:** Multivariable logistic regression analysis examining associations between perceived food allergy and various clinical, atopic, and socioeconomic factors.

Factor	Perceived food allergies			
	Unadjusted OR (95% CI)	*p*	Adjusted OR (95% CI)	*p*
Maternal education ≥ bachelor degree	1.68 (1.47–1.93)	**<.001**	1.70 (1.32–2.17)	**<.001**
Paternal education ≥ bachelor degree	1.41 (1.23–1.62)	**<.001**	1.04 (0.81–1.33)	.773
Older siblings	1.14 (0.99–1.30)	.064	3.77 (0.52–27.22)	.188
No. of older siblings	0.84 (0.75–0.93)	.**001**	1.01 (0.99–1.03)	.235
Younger siblings	1.14 (1.00–1.31)	.054	1.02 (0.81–1.27)	.901
No. of younger siblings	1.06 (0.92–1.21)	.438		
Nursery attendance	0.80 (0.66–0.97)	.**021**	0.92 (0.72–1.19)	.538
Breastfeeding ever	0.95 (0.83–1.01)	.465		
Complementary food before 7 months	1.02 (0.81–1.28)	.896		
Personal history of				
Asthma	0.35 (0.27–0.44)	**<.001**	0.57 (0.40–0.82)	.**003**
Wheeze ever	0.49 (0.42–0.58)	**<.001**	0.80 (0.62–1.02)	.071
Allergic rhinitis	0.40 (0.34–0.45)	**<.001**	0.60 (0.49–0.74)	**<.001**
Atopic dermatitis	0.21 (0.18–0.24)	**<.001**	0.28 (0.23–0.34)	**<.001**
Paternal history of				
Asthma	0.54 (0.41–0.69)	**<.001**	1.12 (0.73–1.72)	.618
Eczema	0.60 (0.50–0.73)	**<.001**	1.41 (1.04–1.91)	.**026**
Rhinitis	0.56 (0.48–0.65)	**<.001**	0.83 (0.68–1.02)	.077
AFR	0.32 (0.26–0.40)	**<.001**	0.48 (0.35–0.67)	**<.001**
Maternal history of				
Asthma	0.52 (0.39–0.68)	**<.001**	0.95 (0.64–1.42)	.806
Eczema	0.46 (0.38–0.54)	**<.001**	0.82 (0.63–1.05)	.119
Rhinitis	0.54 (0.47–0.63)	**<.001**	0.91 (0.73–1.13)	.386
AFR	0.31 (0.26–0.38)	**<.001**	0.45 (0.34–0.60)	**<.001**
Cat ownership	1.31 (0.89–1.92)	.167		
Dog ownership	0.87 (0.68–1.12)	.274		
Farm living	1.31 (0.57–3.01)	.522		
Household smokers	1.13 (0.98–1.31)	.103		
Maternal smoking—now	0.98 (0.75–1.27)	.860		
Maternal smoking during pregnancy	0.99 (0.59–1.66)	.967		

*Note*: The significant values are bolded.

## 4 DISCUSSION

This study represents the first longitudinal analysis of FA trends in young Chinese children, revealing distinct patterns over a 15‐year period. Our data show a significant rise in parent‐reported FA (6.3%–8.6%) alongside stable probable FA prevalence (4.6%–5.4%), suggesting a growing gap between perceived and clinically confirmed cases. Notably, allergic reactions to egg, peanut, tree nuts, and shellfish showed sustained increases, providing valuable insights into the evolving landscape of allergic conditions in the region, highlighting key patterns and shifts over time that enhance understanding of FA within China's unique environmental, dietary, and healthcare context.

Our findings revealed a significant increase in perceived food allergy (parent‐reported) from 6.3% in 2006–2007 to 8.6% in 2020–2021. This rise was interrupted by a slight decline from 9.5% in 2013–2014 to 8.6% in 2020–2021, which may reflect pandemic‐related healthcare avoidance or transient reductions in allergen exposure due to social distancing. The prevalence of FA has reportedly increased, particularly in developed Asian economies and major metropolitan centers, creating a pronounced urban–rural divide with 2–4× higher rates in cities compared to rural areas (e.g., Hong Kong vs. mainland China).^11^ A study in Chongqing found FA prevalence among children ≤2 years increased from 3.5% (1999) to 11.1% (2019), with the 2009–2019 increase not statistically significant (*p* = .086).^12^ These rates appear high compared to challenge‐confirmed FA in Melbourne (10% in 1 year olds).^2^ The inclusion of symptoms like eczema, bloody stools, and diarrhea—commonly associated with non‐IgE‐mediated FA or other pediatric conditions—suggests possible FA overdiagnosis.^13^ Perceived food allergy often leads to the overdiagnosis of true FA due to reliance on subjective symptomatology without corroborative diagnostic testing. Parental over‐reporting, fueled by heightened awareness, societal anxiety, or misattribution of nonallergic reactions (e.g., intolerances), likely contributes to this gap.^14^, ^15^ In Western countries like England, Norway, and Australia, prescriptions for specialized formulas have surged tenfold, significantly surpassing the expected prevalence of cow's milk protein allergy.^16^, ^17^

The rising perceived food allergy prevalence is in contrast to the relatively stable prevalence of probable food allergy (doctor‐diagnosed) (ranging from 4.6% to 5.4%) and food avoidance (ranging from 11.0% to 12.9%) over the years. Pandemic‐related disruptions (e.g., masking, daycare closures) may have temporarily reduced allergen exposure in 2020–2021, potentially dampening both perceived and actual FA incidence. Nonetheless, the steady rate of probable food allergy in HK seems akin to the trend in the West. Peanut allergy prevalence was studied in three Isle of Wight birth cohorts (1989–2002). Peanut sensitization peaked in 1994–1996 (3.3%) compared to 1989 (1.3%) and 2001–2002 (2.0%).^18^ Similarly, clinical peanut allergy rose from 0.5% to 1.4% between 1989 and 1994–1996, then slightly decreased to 1.2% in 2001–2002. Overall, no significant changes in peanut allergy rates were noted between 1994 and 2002. In Melbourne, Australia, two cohort studies involving infants aged 12 months recruited 10 years apart, totaling 7209 participants, spanning 2007–2011 and 2018–2019, showed a slight, nonsignificant decrease in peanut allergy prevalence from 3.1% in 2007–2011 to 2.6% in 2018–2019 (*p* = .26).^19^

Aggregate food allergy prevalence rates potentially mask epidemiological complexity, as stability at the population level obscures marked age‐specific disparities (e.g., rising preschooler incidence) and divergent allergen‐specific trajectories (e.g., egg/peanut surges).

Analysis of English healthcare data (1998–2018) showed probable FA incidence doubled from 75.8 to 159.5 cases per 100,000 person‐years (2008–2018), with prevalence rising from 0.4% to 1.1%.^20^ Importantly, the English data reveal age‐specific variations, with the most dramatic increases seen in preschool children (0–4 years), where prevalence reached 4.63% by 2018, while adult rates remained relatively modest at under 1%. Our study identifies both age‐specific variations and distinct allergen‐specific food‐allergic reactions, with egg reactions peaking at 2 years and shellfish reactions reaching maximum levels by age 6. Notably, while certain allergens, including egg, peanut, tree nuts, and shellfish, showed increasing trends over time, others remained stable, underscoring the heterogeneous nature of food allergy epidemiology. Previous reports highlighted that the traditional rarity of peanut allergies (<1% prevalence) persists in most of Asia, though rates are increasing in Westernized Asian cities.^21^, ^22^ The rising FA prevalence in urbanized regions mirrors Western patterns while maintaining distinct regional features.^23^ Emerging allergens include tree nuts in Japan (with walnut‐induced anaphylaxis increasing 150% from 2017 to 2019) and wheat in Japan/Korea.^23^ The observed temporal age‐specific and allergen‐specific variations emphasize the critical need for stratified diagnostic frameworks. Such targeted approaches are essential for accurately characterizing food allergy epidemiological trends, as they capture the dynamic nature of allergic disease expression across different life stages and geographical areas, providing more precise insights into population‐level patterns.

While probable food allergy prevalence remains stable, the gap with perceived cases suggests a rise in unconfirmed allergies. The rising incidence of anaphylaxis in HK, particularly among children, may, in part, reflect this phenomenon.^7^, ^8^ Our longitudinal analysis identified that allergic reactions to egg and peanut showed the most concerning sustained increases, with prevalence doubling and tripling, respectively, over 15 years. These trajectories mirror global trends linked to modern dietary shifts and environmental factors.^21^ The five‐fold surge in tree nut allergy, particularly post‐2013, aligns with rising consumption of nut‐based products and cross‐reactivity patterns observed in other industrialized nations.^23^, ^24^ Underdiagnosis of evolving FA patterns can exacerbate anaphylaxis risk through delayed recognition. This gap may highlight barriers such as limited access to allergists, uneven distribution of allergy care centers, and insufficient availability of standardized allergy testing services within the region, impeding accurate and timely diagnoses.^13^, ^25^ The COVID‐19 pandemic likely exacerbated these access issues, as pandemic‐related restrictions, including lockdowns and isolation policies, further limited healthcare access and allergist consultations during our study period. Our earlier study revealed that a history of AFR, particularly to eggs and peanuts, was a risk factor for impaired life quality in the affected families.^26^ These findings underline the pressing need to develop a more robust allergy healthcare infrastructure. Efforts should include increasing allergist training programs, expanding standardized diagnostic services, and promoting public education campaigns to bridge the knowledge gap between perceived and actual food allergies.

Divergent trends were observed across allergic conditions: while FA and eczema (from 31.4% to 33.6%, *p* = .014) rose, asthma (from 5.0% to 2.1%, *p* < .001), and wheezing illnesses (from 8.1% to 3.1%, *p* < .001) declined (Table S4). These differences may reflect distinct environmental drivers—urbanization and dietary shifts for FA/eczema versus reduced viral exposures and improved air quality for asthma.^27^ The contrasting trends for different allergic conditions align with findings from other Western countries.^28^ Future studies should explore why FA and eczema resist the protective factors mitigating respiratory allergies.

Food allergy in Chinese preschoolers was associated with higher maternal education (aOR: 1.70), reflecting enhanced health literacy and healthcare‐seeking behaviors. A parental history of AFR similarly increases FA odds through heightened awareness. Moreover, children with existing allergic conditions (asthma, allergic rhinitis, and atopic dermatitis) had lower odds of perceived food allergy, likely due to the increased medical attention leading to more accurate diagnoses. The higher proportion of food‐allergic children born in HK (95.7% vs. 93.9%) and fewer younger siblings (52.5% vs. 57.3%) among affected children support the hygiene hypothesis, suggesting that reduced early‐life microbial exposure in urbanized environments may increase allergic disease susceptibility. Other environmental factors, such as breastfeeding, pet ownership, and nursery attendance, demonstrated no significant associations, emphasizing that early microbial exposure patterns are particularly critical in immune system development and susceptibility to allergic diseases.

A key strength of this study is its role as one of the first population‐based time trend analyses of FA in Asia, utilizing consistent methodology and identical study questionnaires across multiple time points to capture changes in FA prevalence and characteristics among Chinese preschool children, providing valuable insights into regional epidemiology. However, the findings were based on parent‐reported FA, which might lead to overestimation due to the absence of objective diagnostic confirmation such as food‐specific IgE testing or challenge results. Furthermore, our definition of perceived food allergy included parent‐reported reactions occurring ever in the child's lifetime, which captures children whose allergies may have resolved. Without access to the results of allergy tests or evaluations, we could not confirm the accuracy of the parent‐reported diagnoses or distinguish between current and resolved allergies. It has been previously demonstrated that self‐reported diagnoses overestimated the prevalence of FA.^29^ Nevertheless, the consistency in data collection ensures that the observed trends over time, while potentially reflecting an overestimate of absolute prevalence, remain valid for assessing relative change and patterns in FA awareness and concern within the same population. Future studies should incorporate medical record verification or prospective clinical assessment to address the important relationship between anaphylaxis rates and food allergy prevalence trends.

In conclusion, this longitudinal study of 11,537 Chinese children aged 2–7 years revealed a sustained rise in perceived food allergy among young Chinese children (6.3% to 8.6%) from 2006 to 2021, contrasting stable probable food allergy prevalence (4.6%–5.4%). Notably, allergic reactions to egg, peanut, tree nuts, and shellfish increased significantly over 15 years, with peanut and tree nut allergies tripling and rising fivefold, respectively. Concurrently, atopic comorbidities shifted: asthma and wheezing declined, eczema increased, and rhinitis fluctuated. These findings underscore a growing divergence between perceived and confirmed FA rates, alongside evolving allergen‐specific burdens, highlighting the need for targeted public health strategies to address rising allergies and improve diagnostic accuracy.

## AUTHOR CONTRIBUTIONS

**Agnes S. Y. Leung:** Conceptualization; funding acquisition; writing – original draft; investigation; writing – review and editing; methodology; validation; visualization; formal analysis; project administration; data curation; supervision; resources; software. **Oi Man Chan:** Writing – review and editing. **Noelle Anne Ngai:** Writing – review and editing. **Hing Yee Sy:** Writing – review and editing; project administration; data curation. **Ann W. S. Au:** Writing – review and editing; project administration. **Rosetta T. C. Leung:** Project administration; writing – review and editing. **Jaimie Y. M. Ngai:** Writing – review and editing; project administration. **Edmund Yung:** Project administration; writing – review and editing. **Man Fung Tang:** Project administration; writing – review and editing; data curation. **Gary W. K. Wong:** Writing – review and editing. **Ting Fan Leung:** Writing – review and editing; conceptualization; funding acquisition; methodology; investigation; project administration; supervision.

## FUNDING INFORMATION

This study was supported by a research grant from the Hong Kong Institute of Allergy Research Grant and the Direct Grant for Research from The Chinese University of Hong Kong (2021.051). The funders had no role in study design, data collection and analysis, decision to publish, or preparation of the manuscript.

## CONFLICT OF INTEREST STATEMENT

The authors declare that they have no conflict of interest.

## PEER REVIEW

The peer review history for this article is available at https://www.webofscience.com/api/gateway/wos/peer‐review/10.1111/pai.70188.

## Supporting information


Appendix S1.

Tables S1–S6.

## Data Availability

Requests for de‐identified summary data can be made to the corresponding authors.
