# Supplementary material for: Trends in food allergy among Hong Kong preschoolers: Findings from 2006, 2013, and 2020 surveys
Source: Pediatr Allergy Immunol. 2025 Sep 1;36(9):e70188. doi: 10.1111/pai.70188 (PMC12400011; doi:10.1111/pai.70188)
Supplement: Supplementary file 1 — Appendix S1. [file PAI-36-e70188-s001.zip › pai70188-sup-0002-FigureS1-S2@KG supplementary data-27May25_clean.docx]

**Supplementary information**

**Methodology**

The parent-administered questionnaire, comprising 89 items in Chinese, integrated adapted questions from the food allergy (FA) questionnaire developed by Melbourne’s Royal Children’s Hospital Allergy Clinic and questions on asthma/atopic disorder from the Chinese ISAAC (International Study of Asthma and Allergies in Childhood) questionnaire.^S1-3^

**Perceived Food Allergy (Parent-Reported Food Allergy):**Parents were asked whether their child had ever experienced adverse food reactions (AFR) in their lifetime (AFR ever) or within the past 12 months (current AFR). Children with affirmative responses to either question were classified as having perceived food allergy (parent-reported AFR). Supplementary Table 1 details the frequency of food allergic reactions in the past 12 months, categorized as occurring one time, two times, or three times and more, with approximately 40% of all perceived allergic food reactions occurring within the most recent 12-month period. Parents also identified specific foods or ingredients linked to AFR from a predefined list of 32 items (e.g., cow’s milk, soy-based formula) or additional foods they specified. Follow-up questions addressed avoidance practices, severity, and frequency of AFR.

**Probable Food Allergy (Doctor-Diagnosed Food Allergy):**The definition of probable food allergy in this study reflects parent-reported physician confirmation, without independent validation of diagnostic criteria. Children were categorized as having probable food allergy if parents affirmed that a physician had diagnosed their child with AFR. However, the questionnaire did not explicitly verify whether diagnoses were based solely on parental reports of symptoms or included objective testing (e.g., IgE sensitization, oral food challenges). This limitation is acknowledged in the discussion.

**Study population - sampling**

Different institutions participated in the three survey waves (2006, 2011, 2016). The selection of nurseries and kindergartens was randomized across Hong Kong’s four geographic regions (New Territories East, New Territories West, Kowloon, and Hong Kong Island), with respondents proportions intentionally aligned to **reflect the population distribution of children aged 0–14 years** according to the 2006, 2011, and 2016 Hong Kong Census data ("Domestic Households by District Council District and Year"). Supplementary Figure 1 demonstrates that the **respondent percentages largely corresponded to the population distribution** of children in each region for the respective census years. Any minor deviations (e.g., New Territories West’s underrepresentation in 2020) would be contextualized by the multi-year design, population-weighted statistical adjustments, and random selection protocol for educational institutions. The apparent underrepresentation in New Territories West and overrepresentation in Kowloon during 2020 resulted from the random selection process identifying schools with smaller student populations in this region, while simultaneously selecting larger-enrollment institutions in Kowloon. This enrollment size variation accounts for the minor regional deviations observed.

This census-based, proportional sampling methodology yielded several methodological strengths. First, geographic representativeness was achieved by capturing urban-rural variations in healthcare access and specialist availability, while ensuring socioeconomic status (SES) diversity across regions (e.g., higher-SES Hong Kong Island versus lower-SES New Territories West). Second, demographic stability was maintained across phases for race (≥95.6% Chinese, P=.323 for Phase 1 vs. 3) and gender (50.4–52.0% male, P ≥.05 across phases), while age distributions showed shifts reflecting societal trends such as delayed school entry despite stable mean ages (4.37 vs. 4.27 years, P =.441) (Supplementary Table 6). Third, SES trends mirrored real-world development and evolving socioeconomic dynamics intrinsic to Hong Kong's development, with university-educated parents increasing significantly from 12.6% (mothers) and 15.8% (fathers) in 2006–2007 to 44.9% and 44.0% respectively in 2020–2021 (P<.001), corresponding to Hong Kong's expansion of higher education. Additionally, the gradual increase in local-born parents (mothers: 48.2% to 55.6%, P <.001; fathers: 67.8% to 69.8%, P=.021) and children (91.8% to 94.3%, P <.001), aligned with improved healthcare access and socioeconomic mobility.

**Supplementary references**

1. ISAAC Steering Committee. Phase II modules of the International Study of Asthma and Allergies in Childhood (ISAAC). Münster: Institute of Epidemiology and Social Medicine, University of Münster. 1998.
2. Wong GW, Leung TF, Ko FW, et al. Declining asthma prevalence in Hong Kong Chinese schoolchildren. Clin Exp Allergy 2004: 34: 1550–5.
3. Wong GW, Leung TF, Ma Y, Liu EK, Yung E, Lai CK. Symptoms of asthma and atopic disorders in preschool children: prevalence and risk factors. Clin Exp Allergy 2007: 37: 174–9.

**Supplementary Figure Legend:**

Supplementary Figure 1 illustrates that respondent percentages closely aligned with the population distribution of children in each geographic region across the respective census years. Abbreviation: HK: Hong Kong Island, KLN: Kowloon, NTE: New Territories East, and NTW: New Territories West

Supplementary Figure 2 illustrates the distribution of food allergens across age groups.
