# Supplementary figures and images for: Trends in food allergy among Hong Kong preschoolers: Findings from 2006, 2013, and 2020 surveys
Source: Pediatr Allergy Immunol. 2025 Sep 1;36(9):e70188. doi: 10.1111/pai.70188 (PMC12400011; doi:10.1111/pai.70188)

Population Distribution vs. Respondent Distrubtion by Region

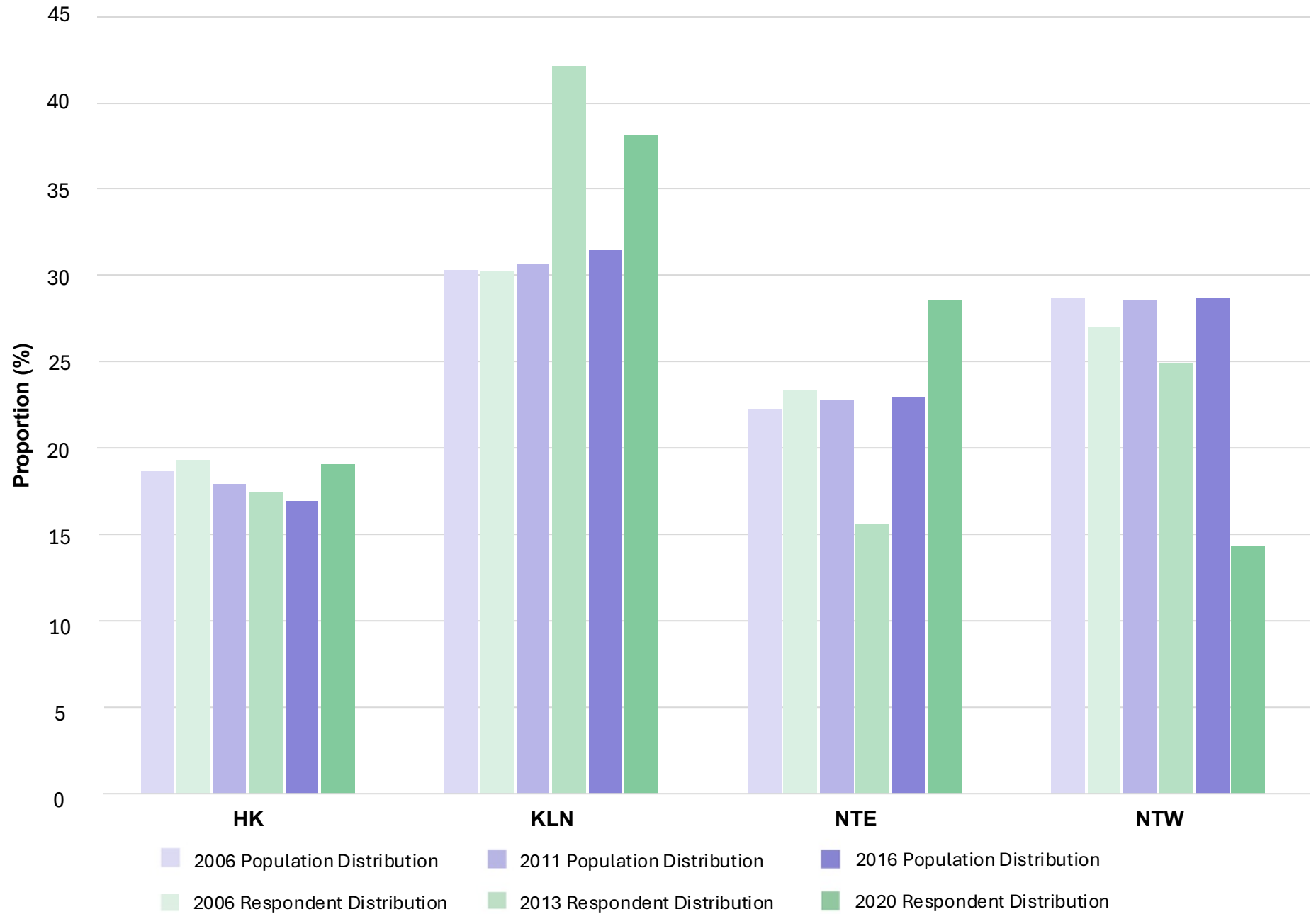

Supplement: Supplementary file 1 — Appendix S1. [file PAI-36-e70188-s001.zip › pai70188-sup-0001-FigureS1@KG Supp Figure 1_20May25_Vf.pdf]

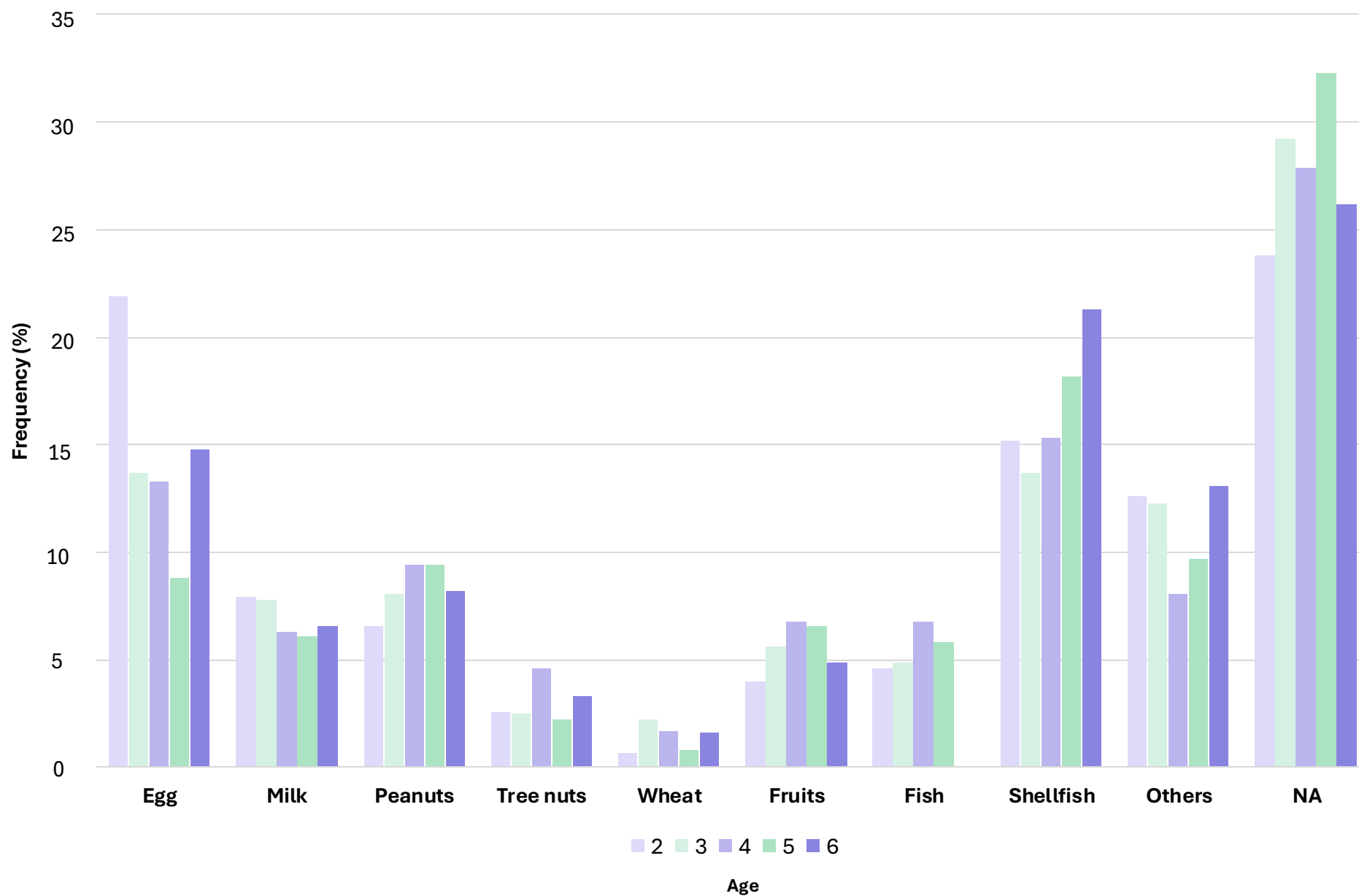

Supplementary Figure 2

Supplement: Supplementary file 1 — Appendix S1. [file PAI-36-e70188-s001.zip › pai70188-sup-0003-FigureS2@KG Supp Figure 2-16Apr25_Vf.pdf]
