# Supplementary material for: Trends in food allergy among Hong Kong preschoolers: Findings from 2006, 2013, and 2020 surveys
Source: Pediatr Allergy Immunol. 2025 Sep 1;36(9):e70188. doi: 10.1111/pai.70188 (PMC12400011; doi:10.1111/pai.70188)
Supplement: Supplementary file 2 — Tables S1–S6. [file PAI-36-e70188-s002.docx]

Supplementary Table 1: Prevalence of allergic conditions among Hong Kong preschool children across three time points (2006–2007, 2013–2014, 2020–2021).

|  | Total populations  Population-weight Frequency % (95% CI) | | | | | |
| --- | --- | --- | --- | --- | --- | --- |
| *Prevalence rates* | **2006-2007** | **2013-2014** | ***P**** | **2020-2021** | ***P^#^*** | **All** |
| *Perceived food allergy*  *(Parent-reported AFR)* | 6.1 (5.4-6.9) | 9.5 (8.6-10.5) | **<.001** | 8.5 (7.7-9.3) | **<.001** | 8 (7.5-8.5) |
| *Probable food allergy*  *(Parent-reported Doctor-diagnosed AFR)* | 4.4 (3.8-5.1) | 4.9 (4.2-5.6) | .385 | 5.1 (4.5-5.8) | .166 | 4.8 (4.4-5.2) |
| *No. of food-allergic reactions*  *in the past 12 months* |  |  |  |  |  |  |
| *One time* | 1.1 (0.8-1.5) | 1.4 (1.1-1.8) | .265 | 1.1 (0.9-1.5) | .262 | 1.2 (1.0-1.4) |
| *Two times* | 0.9 (0.6-1.3) | 0.8 (0.5-1.1) | .462 | 0.9 (0.5-1.1) | .959 | 0.8 (0.7-1.0) |
| *Three times and more* | 1.2 (0.9-1.6) | 1.7 (1.4-2.2) | .098 | 1.4 (1.0-1.7) | .267 | 1.4 (1.2-1.6) |
| *Current food avoidance* | 11.3 (10.3-12.3) | 12.9 (11.9-14.0) | **.028** | 11 (10.1-11.9) | .332 | 11.7 (11.1-12.2) |
| *Atopic co-morbidities* |  |  |  |  |  |  |
| *Asthma* | 5 (4.3-5.7) | 4.6 (3.9-5.3) | .442 | 2.1 (1.7-2.6) | **<.001** | 3.8 (3.5-4.2) |
| *Wheeze in past 12m* | 8.1 (7.3-9.0) | 13.9 (12.8-15.1) | **<.001** | 3.1 (2.6-3.6) | **<.001** | 8 (7.6-8.5) |
| *Rhinitis* | 24 (22.7-25.4) | 32.2 (30.7-33.7) | **<.001** | 24.6 (23.4-25.9) | .264 | 26.7 (26.0-27.5) |
| *Eczema* | 31.4 (29.9-32.9) | 40.2 (38.6-41.8) | **<.001** | 33.6 (32.3-35.0) | **.014** | 34.9 (34.1-35.8) |

*Comparison between 2006-2007 and 2013-2014

#Comparison between 2006-2007 and 2020-2021

Supplementary Table 2: Prevalence of perceived and probable food allergies among Hong Kong preschool children across three time points (2006–2007, 2013–2014, 2020–2021), with associated P-values for trends over time.

| Population-Weight Frequency % (95% Ci) | Perceived food allergy  (Parent-reported AFR) | | | | | Probable food allergy  (Parent-reported Doctor-diagnosed AFR) | | | | |
| --- | --- | --- | --- | --- | --- | --- | --- | --- | --- | --- |
|  | **2006-2007** | **2013-2014** | ***P**** | **2020-2021** | ***P^#^*** | **2006-2007** | **2013-2014** | ***P**** | **2020-2021** | ***P^#^*** |
| *AFR Prevalence* | 6.1 (5.4-6.9) | 9.5 (8.6-10.5) | <.001 | 8.5 (7.7-9.3) | <.001 | 4.4 (3.8-5.1) | 4.9 (4.2-5.6) | .385 | 4.8 (4.4-5.2) | .166 |
| *Atopic co-morbidities* |  |  |  |  |  |  |  |  |  |  |
| *Asthma* | 14.2 (10-19.2) | 10.9 (8-14.6) | .259 | 4.7 (2.9-7.2) | **<.001** | 11.5 (7.2-17.2) | 13.4 (8.9-19.1) | .616 | 5.9 (3.3-9.6) | **.049** |
| *Wheeze ever* | 24.1 (18.9-30) | 33.0 (28.2-38.2) | .023 | 20.1 (16.2-24.5) | .253 | 21.6 (15.8-28.4) | 37.5 (30.6-44.8) | .001 | 24.0 (18.7-29.9) | .585 |
| *Wheeze in past 12m* | 17.0 (12.5-22.3) | 23.4 (19.1-28.1) | .066 | 6.3 (4.2-9.2) | **<.001** | 15.4 (10.5-21.6) | 27.8 (21.6-34.8) | .006 | 5.9 (3.3-9.6) | **.002** |
| *Rhinitis* | 44.2 (37.7-50.9) | 53.6 (48.2-58.9) | **.032** | 42.9 (37.9-48.1) | .761 | 42.4 (34.7-50.3) | 48.8 (41.4-56.3) | .245 | 40.7 (34.2-47.3) | .741 |
| *Eczema* | 67.0 (60.6-72.9) | 72.4 (67.5-77.0) | .170 | 71.7 (66.9-76.2) | .227 | 67.7 (60.1-74.7) | 74.7 (67.9-80.7) | .162 | 69.1 (62.8-75.0) | .777 |
| *Gender* |  |  |  |  |  |  |  |  |  |  |
| *Male* | 51.8 (45.3-58.3) | 53.5 (48.2-58.7) | .688 | 55.8 (50.7-60.9) | .343 | 56.8 (49.1-64.2) | 59.1 (51.7-66.2) | .669 | 62 (55.5-68.2) | .305 |
| *Age, Years* |  |  |  |  |  |  |  |  |  |  |
| *<3* | 11.6 (7.9-16.3) | 12.6 (9.4-16.4) | .731 | 5.8 (3.7-8.5) | **.012** | 13.0 (8.5-18.8) | 10.8 (6.9-16.0) | .538 | 6.8 (4.0-10.7) | .041 |
| *>=3 To <4* | 37.1 (30.9-43.5) | 27.2 (22.7-32.1) | **.013** | 26.7 (22.4-31.4) | **.008** | 31.5 (24.7-38.9) | 26.7 (20.6-33.6) | .334 | 27.6 (22.0-33.8) | .410 |
| *>=4 To <5* | 26.8 (21.3-32.9) | 28.9 (24.3-33.9) | .576 | 33.3 (28.6-38.3) | .095 | 32.1 (25.3-39.6) | 30.1 (23.7-37.2) | .694 | 33.9 (27.9-40.4) | .706 |
| *>=5 To <6* | 23.2 (18.1-29.1) | 27.8 (23.2-32.7) | .226 | 27 (22.6-31.7) | .307 | 22.2 (16.3-29.1) | 29.0 (22.7-36.0) | .156 | 22.6 (17.5-28.5) | .926 |
| *>=6 To <7* | 1.3 (0.4-3.5) | 3.5 (1.9-5.9) | .116 | 7.2 (4.8-10.2) | **.002** | 1.2 (0.3-3.9) | 3.4 (1.4-6.9) | .189 | 8.6 (5.4-12.8) | **.002** |
| *Place Of Birth* |  |  |  |  |  |  |  |  |  |  |
| *Hong Kong* | 96.4 (93.4-98.3) | 96.8 (94.5-98.3) | .819 | 96.7 (94.5-98.2) | .868 | 96.3 (92.5-98.4) | 96.6 (93.1-98.6) | .884 | 95.5 (92.1-97.7) | .691 |
| *Presence of Siblings* |  |  |  |  |  |  |  |  |  |  |
| *Older* | 60.9 (54.3-67.3) | 60.3 (55.0-65.4) | .882 | 33.1 (28.3-38.0) | **<.001** | 55.5 (47.6-63.1) | 60.3 (53-67.4) | .372 | 33.3 (27.3-39.8) | **<.001** |
| *Younger* | 77.3 (71.4-82.5) | 69.7 (64.7-74.5) | .051 | 28.4 (23.9-33.2) | **<.001** | 77.8 (70.9-83.8) | 68 (60.8-74.6) | **.044** | 27.5 (21.9-33.7) | **<.001** |
| *Mother's Education Level* |  |  |  |  |  |  |  |  |  |  |
| *No University* | 77.2 (71.4-82.4) | 42.7 (37.5-48.0) | **<.001** | 50.1 (45-55.3) | **<.001** | 81.5 (75-86.9) | 43.2 (36.0-50.6) | **<.001** | 51.1 (44.6-57.7) | **<.001** |
| *Father's Education Level* |  |  |  |  |  |  |  |  |  |  |
| *No University* | 75.4 (69.5-80.7) | 45.9 (40.7-51.2) | **<.001** | 54.8 (49.7-59.9) | **<.001** | 79.6 (72.9-85.3) | 48.9 (41.5-56.2) | **<.001** | 50.7 (44.1-57.2) | **<.001** |
| *Maternal Allergy History* |  |  |  |  |  |  |  |  |  |  |
| *Asthma* | 3.6 (1.7-6.6) | 9.1 (6.4-12.5) | **.012** | 7.7 (5.3-10.8) | .042 | 2.5 (0.8-5.8) | 11.4 (7.3-16.7) | **.001** | 8.6 (5.4-12.8) | **.013** |
| *Rhinitis* | 33 (27.1-39.4) | 39.2 (34.1-44.4) | .138 | 41.3 (36.3-46.4) | .045 | 31.5 (24.7-38.9) | 36.4 (29.5-43.6) | .344 | 41.2 (34.8-47.7) | .052 |
| *Eczema* | 14.3 (10.2-19.3) | 23.4 (19.1-28.1) | **.008** | 26.7 (22.4-31.4) | **<.001** | 12.3 (8-18.1) | 22.2 (16.5-28.7) | **.018** | 23.1 (17.9-29) | **.008** |
| *AFR* | 17.9 (13.3-23.3) | 14.9 (11.4-19) | .351 | 18.7 (15-23) | .79 | 16.7 (11.5-23) | 14.2 (9.6-19.9) | .531 | 15.4 (11.1-20.6) | .735 |
| *Paternal Allergy History* |  |  |  |  |  |  |  |  |  |  |
| *Asthma* | 3.6 (1.7-6.6) | 6.7 (4.4-9.7) | .107 | 12.1 (9.1-15.8) | **<.001** | 3.7 (1.6-7.5) | 6.8 (3.8-11.3) | .203 | 11.3 (7.6-16) | **.007** |
| *Rhinitis* | 33.9 (28-40.3) | 43.9 (38.7-49.2) | **.018** | 45.7 (40.7-50.9) | **.005** | 28.4 (21.9-35.7) | 43.2 (36-50.6) | **.005** | 44.8 (38.3-51.4) | **.001** |
| *Eczema* | 8.9 (5.7-13.2) | 13.2 (9.9-17.0) | .123 | 25.9 (21.6-30.6) | **<.001** | 12.3 (8-18.1) | 12.5 (8.2-18) | .966 | 20.4 (15.5-26) | **.039** |
| *AFR* | 9.8 (6.4-14.2) | 12 (8.9-15.7) | .423 | 15.2 (11.7-19.1) | .063 | 8 (4.6-13) | 12.5 (8.2-18) | .177 | 14.9 (10.7-20.1) | **.040** |

*Comparison between 2006-2007 and 2013-2014

#Comparison between 2006-2007 and 2020-2021

Supplementary Table 3: Multivariable logistic regression analysis examining associations between probable food allergy and various clinical, atopic, and socioeconomic factors.

|  | **Probable food allergies** | | |  |
| --- | --- | --- | --- | --- |
| **Factor** | **Unadjusted OR (95% CI)** | ***P*** | **Adjusted OR (95% CI)** | ***P*** |
| Maternal education < bachelor’s degree | 1.41 (1.18-1.67) | **<.001** | 1.58 (1.15-2.16) | **.004** |
| Parental education < bachelor’s degree | 1.27 (1.07-1.51) | **.007** | 0.85 (0.62-1.17) | .328 |
| Older siblings | 1.21 (1.02-1.43) | **.032** | 1.13 (0.88-1.47) | .340 |
| No. of older siblings | 1.00 (0.999-1.003) | .292 |  |  |
| Younger siblings | 1.17 (0.99-1.39) | .073 | 1.03 (0.77-1.37) | .861 |
| No. of younger siblings | 0.999 (0.997-1.001) | .545 |  |  |
| Nursery attendance | 0.83 (0.65-1.07) | .145 |  |  |
| Breastfeeding ever | 0.99 (0.83-1.17) | .897 |  |  |
| Complementary food before 7 months | 1.16 (0.88-1.54) | .306 |  |  |
| Personal history of |  |  |  |  |
| Asthma | 0.34 (0.25-0.46) | **<.001** | 0.59 (0.38-0.92) | **.018** |
| Wheeze ever | 0.46 (0.38-0.56) | **<.001** | 0.77 (0.57-1.05) | .103 |
| Allergic rhinitis | 0.48 (0.40-0.57) | **<.001** | 0.72 (0.55-0.93) | **.013** |
| Atopic dermatitis | 0.22 (0.19-0.27) | **<.001** | 0.26 (0.20-0.35) | **<.001** |
| Paternal history of |  |  |  |  |
| Paternal asthma | 0.58 (0.42-0.81) | **.001** | 1.06 (0.62-1.81) | .833 |
| Paternal eczema | 0.67 (0.53-0.86) | **.002** | 1.25 (0.86-1.82) | .245 |
| Paternal rhinitis | 0.65 (0.54-0.79) | **<.001** | 0.91 (0.70-1.18) | .474 |
| Paternal AFR | 0.36 (0.28-0.48) | **<.001** | 0.50 (0.33-0.75) | **<.001** |
| Maternal history of |  |  |  |  |
| Maternal asthma | 0.49 (0.35-0.69) | **<.001** | 0.89 (0.54-1.47) | .654 |
| Maternal eczema | 0.56 (0.45-0.71) | **<.001** | 0.95 (0.69-1.32) | .765 |
| Maternal rhinitis | 0.61 (0.50-0.74) | **<.001** | 1.003 (0.76-1.32) | .985 |
| Maternal AFR | 0.38 (0.29-0.49) | **<.001** | 0.46 (0.33-0.65) | **<.001** |
| Cat ownership | 1.07 (0.68-1.67) | .776 |  |  |
| Dog ownership | 0.96 (0.69-1.33) | .817 |  |  |
| Farm living | 1.53 (0.48-4.86) | .468 |  |  |
| Household smokers | 1.08 (0.90-1.30) | .420 |  |  |
| Maternal smoking - now | 0.955 (0.69-1.33) | .785 |  |  |
| Maternal smoking during pregnancy | 0.96 (0.51-1.83) | .906 |  |  |

Supplementary Table 4: Temporal trends in perceived and probable food-allergic reactions among Hong Kong preschool children (2006-2007, 2013-2014, 2020-2021).

|  | 2006-2007 |  | 2013-2014 |  | 2020-2021 |  |
| --- | --- | --- | --- | --- | --- | --- |
| Food allergy | Perceived, n (%) | Probable, n (%) | Perceived, n (%) | Probable, n (%) | Perceived, n (%) | Probable, n (%) |
| Skin reactions (hives, rash, itching) | 106 (47.3) | 54 (57.4) | 203 (59.4) | 92 (66.7) | 226 (62.3) | 110 (67.1) |
| Swelling | 36 (16.1) | 23 (24.5) | 82 (24.0) | 48 (34.8) | 86 (23.7) | 52 (31.7) |
| Nausea or Vomiting | 23 (10.3) | 13 (13.8) | 29 (8.5) | 12 (8.7) | 24 (6.6) | 14 (8.5) |
| Abdominal Pain | 8 (3.6) | 1 (1.1) | 10 (2.9) | 6 (4.3) | 12 (3.3) | 7 (4.3) |
| Diarrhea | 8 (3.6) | 4 (4.3) | 21 (6.1) | 8 (5.8) | 20 (5.5) | 11 (6.7) |
| Cough | 7 (3.1) | 6 (6.4) | 13 (3.8) | 8 (5.8) | 22 (6.1) | 16 (9.8) |
| Shock | 2 (0.9) | 2 (2.1) | 3 (0.9) | 3 (2.2) | 6 (1.7) | 4 (2.4) |
| Other Reactions | 23 (10.3) | 12 (12.8) | 31 (9.1) | 13 (9.4) | 24 (6.6) | 13 (7.9) |

Supplementary Table 5: Distribution of perceived and probable food allergies by food allergen subgroups across three study phases.

|  | 2006-2007 |  |  |  | 2013-2014 |  |  | 2020-2021 |  |  |  |  |
| --- | --- | --- | --- | --- | --- | --- | --- | --- | --- | --- | --- | --- |
|  | Perceived | Probable | *P* value |  | Perceived | Probable | *P* value | Perceived | Probable |  | *P* value |  |
| Egg | 13.50% | 10.40% | .283 |  | 14.30% | 17.20% | .246 | 13.10% | 15.00% |  | .339 | |
| Milk | 6.20% | 6.20% | 1.000* |  | 9.10% | 11.80% | .131 | 5.40% | 6.00% |  | .687 | |
| Peanuts | 6.60% | 8.80% | .369 |  | 7.70% | 7.30% | .821 | 12.40% | 11.90% |  | .801 | |
| Tree nuts | 1.50% | 2.10% | .704* |  | 2.10% | 1.50% | .548* | 5.80% | 6.00% |  | .911 | |
| Wheat | 0.00% | 0.00% | N/A |  | 1.50% | 2.30% | .783* | 2.60% | 2.50% |  | .929 | |
| Fruits | 3.10% | 3.60% | .749* |  | 9.40% | 6.90% | **.038** | 6.60% | 5.30% |  | .411 | |
| Fish | 4.60% | 4.10% | .791* |  | 7.90% | 8.40% | .804 | 5.60% | 5.30% |  | .867 | |
| Shellfish | 21.20% | 16.10% | **.046** |  | 16.00% | 11.50% | **.046** | 15.70% | 13.20% |  | .246 | |
| Others | 11.20% | 7.80% | **.042** |  | 9.40% | 9.50% | .999 | 11.40% | 9.70% |  | .340 | |
| NA | 32.00% | 40.90% | **.034** |  | 22.80% | 23.70% | .752 | 21.50% | 25.10% |  | .158 | |
| *by Fisher’s exact test  NA (not available/ unknown) | |  |  |  |  |  |  |  |  |  |  |  |

Supplementary Table 6 : Temporal Changes in Demographic and Socioeconomic Characteristics of Participants Across Three Survey Phases (2006–2007, 2013–2014, 2020–2021)

| Population-Weight Frequency % (95% Ci) | 2006-2007 | 2013-2014 | | *P* value between Phase 1&2 | | 2020-2021 | | *P* value  between Phase 2&3 | | *P* value  between Phase 1&3 | |  |
| --- | --- | --- | --- | --- | --- | --- | --- | --- | --- | --- | --- | --- |
| Ethnicity |  | |  | | <.001 | |  | | <.001 | | .323 | |
| Chinese | 95.6 (94.9-96.2) | | 97.7 (97.2-98.2) | |  | | 96.0 (95.4-96.6) | |  | |  | |
| Non-Chinese | 1.4 (1.1-1.8) | | 0.9 (0.7-1.3) | |  | | 2.3 (1.9-2.8) | |  | |  | |
| Male | 50.4 (48.8-52.0) | | 50.4 (48.8-52.0) | | .955 | | 52.0 (50.5-53.5) | | .145 | | .158 | |
| Age, mean (95% C.I.) | 4.37 (4.14-4.59) | | 4.10 (3.92-4.28) | | .070 | | 4.27 (4.12-4.41) | | .152 | | .441 | |
| *<3* | 7.0 (6.3-7.9) | | 11.3 (10.3-12.3) | | <.001 | | 6.8 (6.1-7.6) | | <.001 | | .723 | |
| *>=3 To <4* | 32.6 (31.1-34.1) | | 30.8 (29.3-32.3) | | .084 | | 27.4 (26.1-28.7) | | <.001 | | **<.001** | |
| *>=4 To <5* | 30.9 (29.5-32.4) | | 28.3 (26.9-29.8) | | .015 | | 30.6 (29.2-21.9) | | .029 | | .732 | |
| *>=5 To <6* | 26.7 (25.3-28.1) | | 26.4 (25.0-27.8) | | .756 | | 26.8 (25.5-28.2) | | .631 | | .872 | |
| *>=6 To <7* | 2.2 (1.8-2.8) | | 3.0 (2.5-3.6) | | .047 | | 7.8 (7.1-8.7) | | <.001 | | **<.001** | |
| Child born in HK | 91.8 (90.9-92.6) | | 95.2 (94.4-95.8) | | <.001 | | 94.3 (93.6-95.0) | | .087 | | **<.001** | |
| Mother born in HK | 48.2 (46.7-49.8) | | 56.3 (54.6-57.8) | | <.001 | | 55.6 (54.1-57.0) | | .539 | | **<.001** | |
| Father born in HK | 67.8 (66.3-69.2) | | 70.4 (68.9-71.9) | | .007 | | 69.8 (68.5-71.2) | | .580 | | **.021** | |
| Mother’s University education | 12.6 (11.6-13.7) | | 42.8 (41.2-44.4) | | <.001 | | 44.9 (43.5-46.4) | | .058 | | **<.001** | |
| Father’s University education | 15.8 (14.6-16.9) | | 44.7 (43.1-46.3) | | <.001 | | 44.0 (42.6-45.5) | | .573 | | **<.001** | |
